# Taurine Ameliorates Streptozotocin-Induced Diabetes by Modulating Hepatic Glucose Metabolism and Oxidative Stress in Mice

**DOI:** 10.3390/metabo12060524

**Published:** 2022-06-06

**Authors:** Shigeru Murakami, Kohei Funahashi, Natsuki Tamagawa, Ma Ning, Takashi Ito

**Affiliations:** 1 Department of Bioscience and Biotechnology, Fukui Prefectural University, 4-1-1 Matsuoka Kenjyojima, Eiheiji 910-1195, Fukui, Japan; kouhei19950314@gmail.com (K.F.); s1321027@g.fpu.ac.jp (N.T.); tito@fpu.ac.jp (T.I.); 2 Division of Health Science, Graduate School of Health Science, Suzuka University of Medical Science, 1001-1, Kishioka, Suzuka 510-0293, Mie, Japan; maning@suzuka-u.ac.jp

**Keywords:** diabetes, taurine, GLUT-2, oxidative stress, glycogen, glucose metabolism

## Abstract

Taurine is a sulfated amino acid derivative that plays an important role in maintaining the cell function of the living body. Although taurine has been shown to ameliorate diabetes, its mechanism of action has not yet been fully elucidated. The present study investigated the effects of taurine on diabetes focusing on glucose metabolism and oxidative stress. Type 1 diabetes was induced by the administration of streptozotocin (STZ) to male C57BL/6J mice. Taurine was dissolved in drinking water at 3% (*w/v*) and allowed to be freely ingested by diabetic mice. The weight and blood glucose levels were measured weekly. After nine weeks, mice were sacrificed and their serum, liver, and kidney were removed and used for biochemical and histological analyses. A microarray analysis was also performed in normal mice. Taurine alleviated STZ-induced hyperglycemia and hyperketonemia, accompanied by the suppression of the decrease in hepatic glycogen and upregulation of the mRNA expression of hepatic glucose transporter GLUT-2. Furthermore, STZ-induced elevation of oxidative stress in the liver and kidney was suppressed by taurine treatment. These results showed that taurine ameliorated diabetes and diabetic complications by improving hepatic glucose metabolism and reducing oxidative stress.

## 1. Introduction

Taurine, a sulfur-containing amino acid derivative, is abundant in the body of mammals and plays a pivotal role in maintaining cellular homeostasis, such as through osmotic pressure regulation, protein stabilization, antioxidant and anti-inflammatory actions, and calcium ion regulation [1,2]. Taurine is also involved in basic vital activities such as fetal development [3], the maintenance of the organ function, and metabolism [4,5,6,7,8]. Many experiments using animal models and cultured cells have demonstrated that taurine supplementation can suppress the development of a wide range of diseases [9,10,11,12,13]. The important role of taurine in the body has also been demonstrated in taurine transporter knockout mice, in which the tissue taurine concentration is markedly decreased due to the inhibition of the cellular taurine uptake [14]. These mice exhibit a deteriorated organ function and accelerated aging [15,16,17]. 

Among the many disease-preventing effects of taurine, anti-diabetic effects have been reported in animal models and humans [18,19]. In streptozotocin- or alloxan-induced type 1 diabetic animal models, taurine suppresses the development of diabetes [20] and diabetic complications [21,22]. Anti-diabetic effects of taurine have also been demonstrated in type 2 diabetic models, including fructose- or high-fat diet-induced animals and Otsuka Long-Evans Tokushima Fatty (OLETF) rats [23,24]. 

Taurine exhibits hypoglycemic, insulin-sensitizing, and insulin secretagogue activities. Although most of the beneficial effects of taurine on type 1 diabetes have been attributed to its protective effect on pancreatic β-cells, other potential actions have been postulated as anti-diabetic mechanisms of taurine. Taurine regulates the K_ATP_ channel and enhances K^+^-induced depolarization in pancreatic β-cells, which leads to increased insulin secretion [25]. Taurine also increases the islet Ca^2+^ uptake in response to glucose and stimulates insulin release [26]. In addition, taurine elevates extracellular glucose concentrations thorough the glucose transporter GLUT-2 in β-cells and thereby increases insulin secretion [27]. Thus, taurine directly affects pancreatic β-cells and stimulates insulin secretion. 

In addition to the direct effects on pancreatic β-cells, taurine has been shown to affect the insulin target organs, such as liver and skeletal muscle, and ameliorate hyperglycemia and insulin resistance. Taurine improves fatty acid-induced hepatic insulin resistance by modulating the insulin signaling pathway in rats [28]. In the liver and skeletal muscle, taurine enhances the glucose uptake, which is related to amelioration of hyperglycemia and insulin resistance [29]. In vitro and in vivo studies suggest that the hypoglycemic effects of taurine may be mediated through the interaction of taurine with insulin receptor [30]. Taurine supplementation increases both basal and insulin-stimulated tyrosine phosphorylation of the insulin receptor in skeletal muscle and liver [27]. In obese ob/ob mice, taurine improves insulin resistance by activating AMP-activated protein kinase (AMPK) in skeletal muscle [31]. 

Thus, several mechanisms are considered to be involved in anti-diabetic effects. The liver plays a central role in glucose metabolism and is important in the development of diabetes, but few studies have investigated the action of taurine. In this study, we focused on the glucose metabolism and oxidative stress and investigated the effect of taurine on the development of diabetes. 

## 2. Results

### 2.1. Body Weight and Blood Levels of Glucose, Insulin, and Ketone Body 

**Figure 1:**
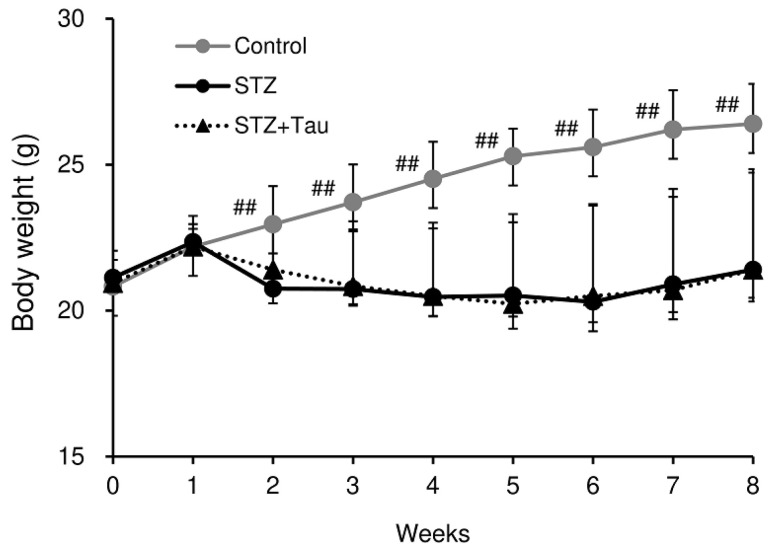
Changes in body weight in control, diabetic, and taurine-treated groups. Each value is expressed as the mean ± S.D. (*n* = 8–12), ## *p* < 0.01 vs. STZ group.

**Figure 2:**
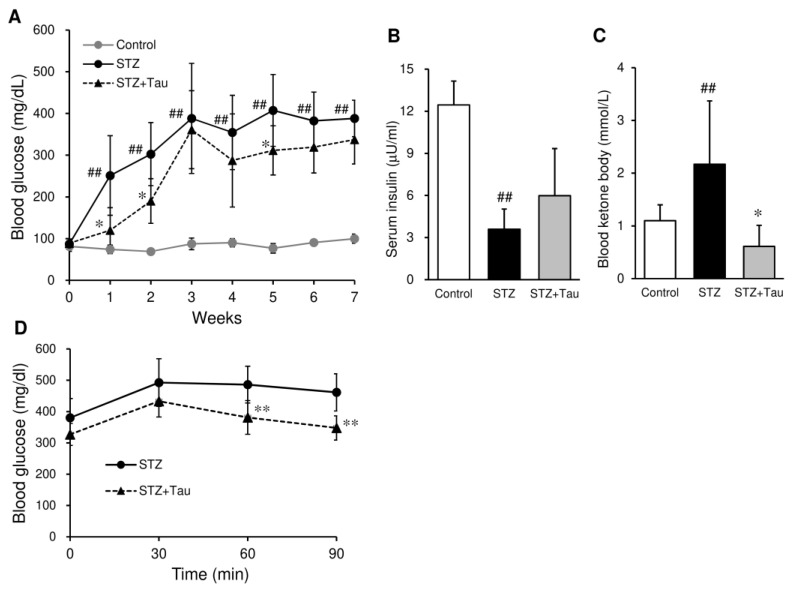
Effects of taurine supplementation on the levels of blood glucose, insulin, and ketone body as well as insulin resistance in diabetic mice. Blood glucose was measured weekly after blood was drawn from the tail vein (**A**). Serum levels of insulin (**B**) and ketone body (**C**) were measured at week 8. Insulin resistance was assessed using an intraperitoneal glucose tolerance test at week 7 (**D**). Each value is expressed as the mean ± S.D. (*n* = 7–12), ## *p* < 0.01 vs. Control group, * *p* < 0.05, ** *p* < 0.01 vs. STZ group.

Control mice gained body weight steadily, but the body weight gain of STZ-treated diabetic mice was completely suppressed (Figure 1). Taurine treatment had no significant effect on food intake and body weight throughout the experimental period. The blood glucose levels in STZ diabetic mice increased until the third week, and then remained around 400 mg/dL (Figure 2A). Taurine treatment significantly suppressed the increase in blood glucose levels at the first, second, and fifth weeks. In other weeks, blood glucose levels were also lower in taurine-treated mice than in diabetic mice, but the difference was not significant. Serum insulin levels were markedly decreased in STZ diabetic mice (Figure 2B). Taurine treatment partially recovered the decrease in insulin levels, but the effect was not significant. Blood levels of ketone body were doubled in STZ mice compared with those of control mice (Figure 2C), and taurine treatment significantly suppressed the elevation of blood ketone body levels.

### 2.2. Insulin Resistance

A glucose tolerance test was performed to evaluate the effect of taurine treatment on insulin resistance. Glucose challenge elevated the blood glucose levels, which remained high in STZ mice (Figure 2D). Blood glucose levels of taurine-treated mice were significantly lower than those in the STZ group at 60 and 90 min after glucose loading.

### 2.3. Glycogen Levels of the Liver and Kidney

**Figure 3:**
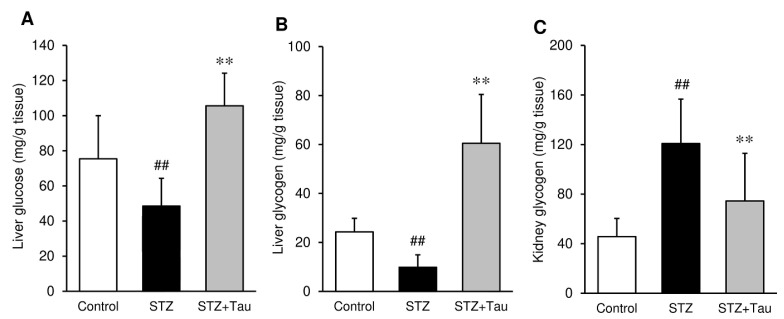
Effects of taurine supplementation on the glucose and glycogen content in the liver and kidney in diabetic mice. (**A**) Liver glucose, (**B**) Liver glycogen, (**C**) Kidney glycogen. Glucose and glycogen levels were measured using commercially available kits. Each value is expressed as the mean ± S.D. (*n* = 7–8). ## *p* < 0.01 vs Control group, ** *p* < 0.01 vs. STZ group.

Glucose and glycogen content were measured in the liver and kidney. The amounts of glucose and glycogen in the liver of STZ mice were significantly lower than those of control mice (Figure 3A,B). In contrast, the glycogen content in the kidney was significantly increased by STZ administration (Figure 3C). These STZ-induced alterations of glycogen and glucose content were significantly recovered by taurine treatment.

### 2.4. MRNA Expression of Glucose Metabolism-Related Genes in the Liver

**Figure 4:**
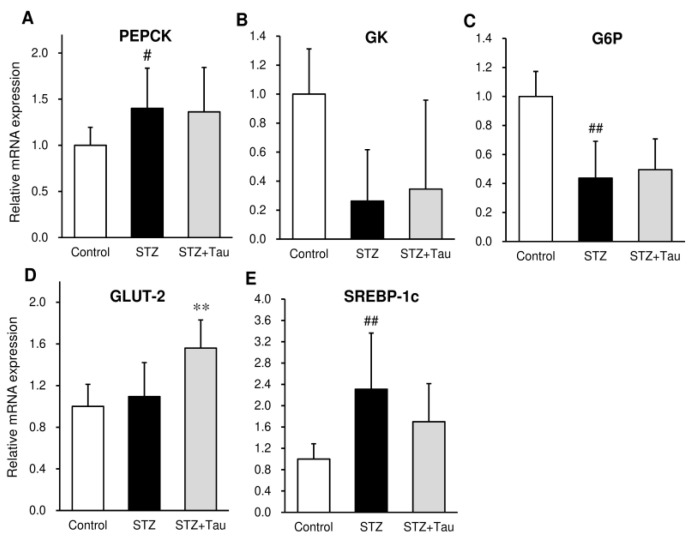
Effects of taurine supplementation on the mRNA expression of glucose metabolism-related genes in the liver of diabetic mice. Total RNA was extracted, and the mRNA expression was assessed using RT-PCR. (**A**) Phosphoenolpyruvate carboxykinase (PEPCK), (**B**) glucokinase (GK), (**C**) glucose-6-phosphatase (G6P), (**D**) glucose transporter-2 (GLUT-2), (**E**) sterol regulatory element-binding protein 1c (SREBP-1c). Each value is expressed as the mean ± S.D. (*n* = 4), # *p* < 0.05, ## *p* < 0.01 vs. Control group, ** *p* < 0.01 vs. STZ group.

To examine the effect of taurine on glucose metabolism, the expression of glucose and lipid metabolism-related genes was investigated in the liver. STZ mice showed a higher mRNA expression of phosphoenolpyruvate carboxykinase (PEPCK) and sterol regulatory element-binding protein 1c (SREBP-1c) than control mice (Figure 4A,E), but a lower mRNA expression of glucokinase (GK) and glucose-6-phosphatase (G6P) (Figure 4B,C). Although the expression of glucose transporter-2 (GLUT-2) was not affected by STZ, taurine treatment significantly increased the GLUT-2 mRNA expression (Figure 4D).

### 2.5. Microarray Analyses

**Figure 5:**
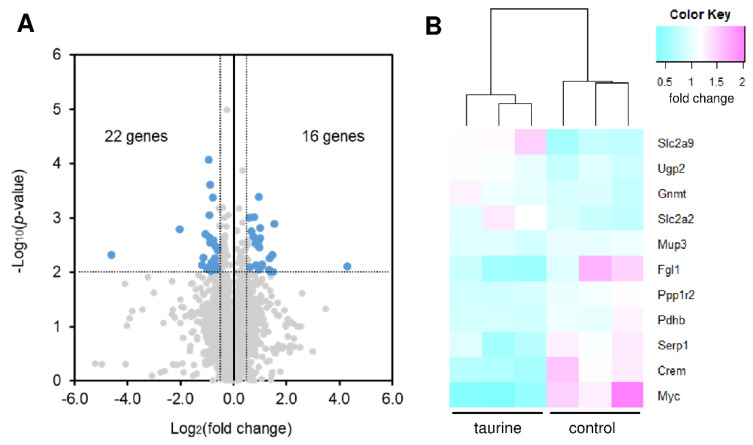
Microarray analysis in the liver from taurine-treated mice. (**A**) Volcano plots showing the differential expression patterns between taurine-treated and control groups. (**B**) Heat maps showing the differentially expressed genes (*p* < 0.05) annotated with GO term for glycogen biosynthesis (GO: 0005978), glucose metabolic process (GO: 0006006), UDP-glucose metabolic process (GO: 0006011) and glucose transport (GO: 0046323) in the liver between taurine-treated and control mice.

**Figure 6:**
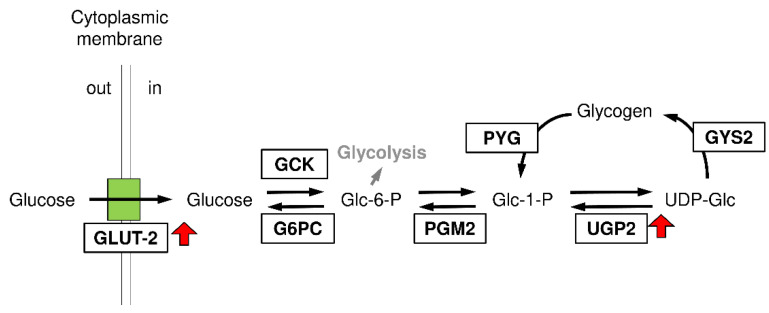
Cellular pathways of glycogen synthesis stimulation by taurine. G6PC: Glucose 6-phosphatase; GCK: Glucokinase; PGM2: Phosphoglucomutase 2; UGP2: UDP-glucose pyrophosphorylase 2; GYS2: Glycogen synthase 2; PYG: Glycogen phosphorylase.

To further evaluate the effect of taurine treatment on the expression of genes responsible for glycogen accumulation in the liver, a microarray analysis was performed on normal mice. Initially, we focused on the differentially expressed genes (DEGs) defined as genes with a fold change of more than 1.5 or less than 0.67 in taurine-treated group compared to the control group and the *p*-value was less than 0.01 in the student’s t-test (Figure 5A). A total of 38 genes were detected as DEGs (16 upregulated and 22 downregulated genes (Table 1). Based on the restrictive false discovery rate analysis (Benjamini and Hochberg’s method), only one gene was listed as a differentially expressed gene (Slc25a38) in taurine-treated mice; the genes associated with glycogen deposition were not detected (Figure 5). Therefore, we focused on the genes annotated with gene ontology terms for glycogen biosynthesis, glucose metabolic process, UDP-glucose metabolic process and glucose transport (Figure 5). Consistent with the gene expression analysis in STZ-induced diabetic mice, GLUT-2 (Slc2a2) was increased by 1.3-fold in taurine-treated mice. In addition, the expression of UDP-glucose pyrophosphorylase 2 (Ugp2), glycine N-methyltransferase (Gnmt), and GLUT-9 (Slc2a9) was increased, while the expression of the genes responsible for the regulation of the glucose metabolic process, such as transcription factors (Myc, Crem) and phosphatase (Ppp1r2), was decreased in the taurine-treated mice. These results suggest that upregulation of GLUT2 and UGP2 may be involved in taurine-induced glycogen increase in the liver (Figure 6).

### 2.6. Oxidative Stress in the Liver and Kidney

**Figure 7:**
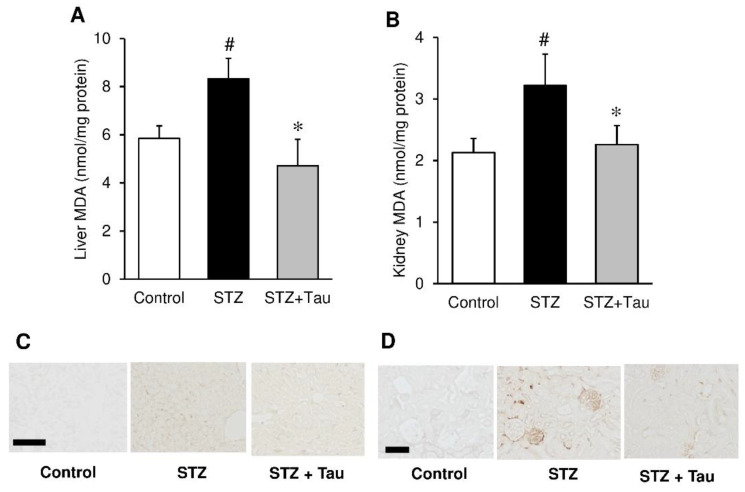
Effects of taurine supplementation on tissue oxidative stress in diabetic mice. MDA levels in liver (**A**) and kidney (**B**) were measured as an oxidative stress marker. Immunostaining 8-OHdG was performed in the liver (**C**) and kidney (**D**). Each value is expressed as the mean ± S.D. (*n* = 7–8), # *p* < 0.05 vs Control group, * *p* < 0.05 vs. STZ group. Scale bar = 100 μm.

The effects of taurine supplementation on oxidative stress were determined in the liver and kidneys. The levels of malondialdehyde (MDA), a marker of lipid peroxidation, were elevated in the liver and kidneys of diabetic mice compared to those of normal mice (Figure 7A,B). Taurine supplementation suppressed the increase in MDA. Consistent with these results, staining with 8-hydroxy-2′-deoxyguanosine (8-OHdG) antibody, a marker of oxidative DNA damage, was increased in the liver and kidneys of STZ mice, but decreased in these organs upon taurine treatment (Figure 7C,D).

### 2.7. Liver Taurine Content

**Figure 8:**
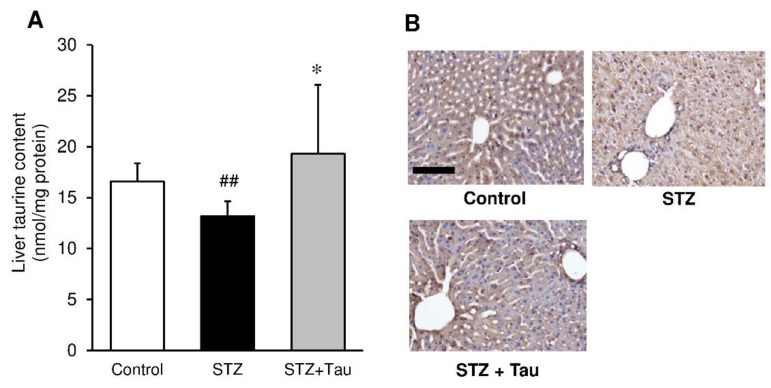
Effects of taurine supplementation on liver taurine content in diabetic mice. Taurine was extracted from the liver and then determined using HPLC (**A**). The liver tissue was fixed with formalin and stained with an anti-taurine antibody (**B**). Each value is expressed as the mean ± S.D. (*n* = 7–8), ## *p* < 0.01 vs Control group, * *p* < 0.05 vs STZ group. Scale bar = 100 μm.

Although the liver taurine content of diabetic mice was significantly lower than that of control mice, taurine treatment restored the reduction in taurine (Figure 8A). Immunohistochemical analyses using anti-taurine antibody supported this finding (Figure 8B).

### 2.8. Pancreatic Insulin and Taurine

**Figure 9:**
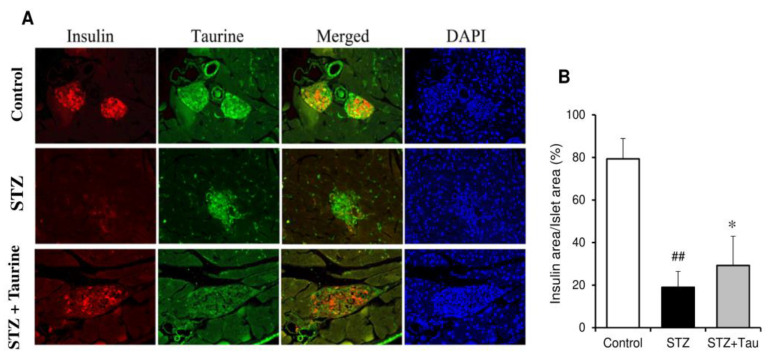
Effects of taurine supplementation on the insulin and taurine levels in pancreas of diabetic mice. Double immunofluorescence staining for taurine and insulin was performed in the pancreas (**A**). The areas of insulin-positive cells were measured, and the percentage in the islets was calculated (**B**). Each value is expressed as the mean ± S.D. (*n* = 24), ## *p* < 0.01 vs Control group, * *p* < 0.05 vs. STZ group.

Localization of taurine and insulin in the pancreas was analyzed using immunofluorescent double staining. The results showed colocalization of taurine (green) with insulin (red) in β-cells of pancreatic islet (Figure 9A). Pancreatic islets of STZ diabetic mice were characterized by a lower β-cell percentage and lower β-cell immunoreactivity for insulin than in control mice. The insulin area was also markedly reduced in STZ diabetic mice (Figure 9B). The intensity of insulin immunoreactivity and area of insulin were increased by taurine supplementation.

## 3. Discussion

The present results showed that taurine treatment ameliorated hyperglycemia and insulin resistance in STZ-induced diabetic mice. The hypoglycemic effect of taurine was mild, and in some weeks, there were no significant differences in blood glucose compared with diabetic mice. There have been conflicting reports concerning the hypoglycemic effect of taurine, depending on the animal models used and the experimental conditions [32,33]. The present biochemical data of the liver and blood suggest that the anti-diabetic effect of taurine may be associated with improvement of hepatic glucose metabolism. 

Glucose is liberated from dietary carbohydrate by hydrolysis within the small intestine and then absorbed into the blood. Elevated concentrations of glucose in the blood stimulate the release of insulin from pancreatic β-cells, and insulin acts on cells to stimulate the uptake, utilization, and storage of glucose, which results in a decrease in blood glucose levels. The liver plays a major role in the modulation of glucose homeostasis by controlling various pathways of glucose metabolism. Hepatic glucose production is primarily regulated by phosphoenolpyruvate carboxykinase (PEPCK) and glucose-6-phosphatase (G6P), which are the rate-limiting enzymes in gluconeogenesis [34]. An analysis of the hepatic mRNA expression related to glucose synthesis, including PEPCK and G6Pase, showed that taurine treatment had no significant effects on the mRNA expression of these enzymes, suggesting that gluconeogenesis is not responsible for the anti-diabetic effect of taurine. In contrast, the mRNA expression of hepatic GLUT-2 was increased by taurine treatment. GLUT-2 is the major type of glucose transporter responsible for the hepatic glucose uptake and utilization [35]. Glucose enters hepatocytes via GLUT-2 and is phosphorylated by glucokinase (GK) and then used to synthesize glycogen. A microarray analysis using normal mice showed that taurine increased the expression of GLUT-2 as well as UGP2, an enzyme involved in glycogen synthesis. These results indicate that taurine has the capacity to promote the uptake of glucose and glycogen synthesis in the liver. In type 1 diabetes, insulin deficiency reduces the hepatic glucose uptake, resulting in hepatic glucose deficiency. However, taurine can normalize hepatic glucose metabolism through the upregulation of the glucose uptake and glycogen synthesis in the liver.

Insulin secretion from β-cells is reduced in type I diabetes, leading to a deficiency of glucose and glycogen levels in the liver. As a result, the hepatic ketone body production from the oxidation of fatty acids increases and is used peripherally as an energy source, which results in diabetic ketoacidosis [36,37]. The levels of blood ketone body were twice as high in STZ diabetic mice as in normal mice in the present study. Consistent with the recovery of hepatic glycogen levels, blood ketone body levels were significantly reduced by taurine treatment. Thus, the suppression of serum ketone body levels by taurine reflects the improvement of glucose metabolism and glycogen content in the liver.

The hypoglycemic effect of taurine in type I diabetic models has been mainly explained by the β-cell protective action of the pancreas and the subsequent suppression of the decrease in insulin secretion [38,39]. The insulin levels of serum and pancreas were markedly decreased in STZ diabetic mice compared to normal mice. Taurine administration to STZ diabetic mice recovered serum insulin levels, but not to a significant degree. In contrast, a histochemical analysis indicated that taurine treatment significantly recovered the area of pancreatic insulin. These data indicate that taurine has a weak β-cell -protective effect. Therefore, improvement of hepatic glucose metabolism by taurine is mainly explained by its stimulatory action on the glucose uptake and subsequent glycogen synthesis in the liver, as shown above, but its protective effect on pancreatic β-cells may also be involved. 

The administration of STZ or alloxane causes diabetic complications due to chronic hyperglycemia. It is well established that reactive oxygen species (ROS) generation or oxidative stress plays an important role in the onset and development of diabetic complications [40,41]. Taurine has been reported to attenuate diabetic complications in STZ- or alloxane-induced diabetic animals, including nephropathy, retinopathy, neuropathy, and cardiovascular problems [42,43]. Taurine has also been shown to suppress liver and kidney injuries caused by toxic substances, including ethanol [44], acetaminophen [45], and arsenic [46]. It is known that the protective effect of taurine on the development of diabetes, diabetic complications, and tissue injuries are closely associated with the reduction of oxidative stress. We previously showed that taurine treatment suppressed the high-fat-diet-induced reduction in the enzyme activity and molecules involved in hepatic anti-oxidant defense, including superoxide dismutase, catalase, and glutathione [47]. In type I diabetes, hepatic glycogen content is decreased due to deficiency of insulin. In contrast, kidney glycogen content is increased. While kidneys usually contain low amounts of glycogen, large deposits of glycogen are present in diabetic kidneys [48]. One of the hallmarks of renal damage in STZ diabetic mice is the accumulation of glycogen [49,50]. Liver glycogen is required for normal energy production, but excessive glycogen accumulation in the kidney impairs the renal function. In the present study, although the kidney glycogen content was elevated in STZ diabetic mice, taurine treatment suppressed this elevation of glycogen levels, suggesting a suppressive effect of taurine on diabetic complications. Consistent with previous findings, biochemical and histochemical data revealed that taurine reduced oxidative stress, as evidenced by the reduction in MDA content and immunostaining of 8-OHdG in both liver and kidney of diabetic mice. In addition, immunofluorescence staining of insulin in the pancreas indicated that taurine protected pancreatic β-cells from toxicity induced by STZ. The cytotoxic action of STZ is mediated by ROS. As oxidative stress is involved in the induction of insulin resistance [28] and β-cell dysfunction, the anti-oxidative action of taurine may be related to not only suppression of diabetic complications, but also amelioration of insulin resistance and hyperglycemia. It is known that hyperglycemia increases tissue oxidative stress by promoting ROS production, which activates NF-κB and induces cellular apoptosis [51]. In fact, taurine has been reported to suppress inflammation and apoptosis in a diabetic model, suggesting that taurine’s anti-diabetic activity is associated with these effects in addition to its antioxidant activity [33].

The present study showed that liver taurine content was reduced in STZ diabetic mice compared to control mice. Immunofluorescence staining showed that pancreatic taurine was also reduced in STZ diabetic mice. Given the important role of taurine in the body, including its antioxidant and osmoregulatory actions, a decrease in taurine in the pancreas may be associated with pancreatic β-cell damage and decreased insulin secretion. In contrast, taurine supplementation to diabetic mice increased pancreatic taurine levels and restored insulin secretion. These findings suggest that taurine plays a role in protecting cells from oxidative damage and suppressing functional deterioration in diabetic mice. Similar reductions in tissue taurine levels have been previously reported in diabetic animals and humans. Indeed, plasma and platelet taurine levels were significantly lower in diabetic patients than in healthy subjects [52]. Taurine levels in the liver were reduced in alloxan-induced diabetic rats [53]. We previously showed that taurine treatment suppressed the high-fat-diet-induced reduction of the enzyme activity and molecules involved in hepatic anti-oxidant defense [47]. Based on these data, reduced liver taurine levels in STZ diabetic mice may be involved at least in part in the disturbance of the glucose metabolism and anti-oxidant defense system in the liver.

The present study and previously reported results suggest that compounds and food components with antioxidant and anti-inflammatory properties may be effective in preventing the progression of diabetes and diabetic complications. Some naturally occurring compounds, such as flavonoids, exhibit antidiabetic effects through their antioxidant and anti-inflammatory actions [54,55]. Thus, taurine is expected to be effective in preventing diabetes and other diseases caused by abnormal glucose metabolism.

## 4. Materials and Methods

### 4.1. Experimental Animals and Treatment

Six-week-old male C57BL/6J mice weighing 19–21 g were purchased from CLEA Japan Inc. (Tokyo, Japan) and housed under a 12-h light/dark cycle at 22 ± 2 °C and 50% ± 5% humidity. The mice had free access to food and water. After 1 week of acclimation, mice were randomly divided into the following three groups of 12 animals each: normal group with citrate buffer as a vehicle control, STZ-treated diabetic group (STZ) and STZ plus taurine group (STZ + Tau). Diabetic mice were given a single intraperitoneal injection of 200 mg/kg STZ (Sigma-Aldrich Inc., St. Louis, MO, USA). STZ was dissolved in citrate buffer at pH 4.5 and used within 20 min of preparation. Induction of diabetes was confirmed by measuring the glucose levels in blood obtained from the tail vein. Taurine was dissolved in drinking water at 3% (*w/v*) and allowed to be freely ingested by mice. Animals were allowed free access to diet and drinking water. The body weight and food intake were monitored every other day. The blood glucose levels were measured once a week using a blood glucometer Nipro Stat Strip (Nipro, Osaka, Japan). Nine weeks after STZ injection, mice were deprived of food overnight, and blood samples were withdrawn from the ophthalmic vein under a mixed anesthetic agent (0.3 mg/kg of medetomidine, 4.0 mg/kg of midazolam, and 5.0 mg/kg of butorphanol; Fujifilm Wako Pure Chemical Co., Osaka, Japan). Liver and kidney tissue were dissected out, weighed, frozen in liquid nitrogen, and stored −80 °C. All of procedures were performed in accordance with the ARRIVE guidelines.

### 4.2. Glucose Tolerance Teat

The mice were fasted overnight and were intraperitoneally injected with glucose solution (2 g/kg body weight). The blood samples were collected from the tail vein of the mice and glucose levels were measured at 0, 30, 90 min after injection using a blood glucometer Nipro Stat Strip (Nipro, Osaka, Japan).

### 4.3. Biochemical Measurements

Serum was obtained by centrifuging at 3000 rpm for 15 min at 4 °C. The serum insulin levels were determined using a commercial ELISA kit (Morinaga Institute of Biological Science, Yokohama, Japan). Blood ketone body concentrations were measured with a blood glucometer Nipro Stat Strip (Nipro) using a dedicated β-hydroxybutyrate test strip.

### 4.4. Glucose and Glycogen Determination of Liver and Kidney

Small pieces of the liver and kidney were homogenized with 10 times the amount of ice-cold 50 mM Tris-HCl buffer (pH 7.4). The homogenate was centrifuged at 15,000× g for 20 min at 4 °C, and the supernatant was used to measure glucose and glycogen levels. The glucose and glycogen content was determined using a commercially available kit; the Glucose Colorimetric/Fluorometric Assay Kit and Glycogen Colorimetric Assay Kit II (Bio Vison, Waltham, MA, USA), respectively, according to the manufacturer’s instructions.

### 4.5. Malondialdehyde (MDA) Determination of Liver and Kidney

MDA was determined as a marker of lipid peroxidation. The supernatant of tissue homogenates as shown above was used to measure MDA levels. The amount of MDA in the liver and kidneys was determined using a commercially available kit; the Malondialdehyde Assay Kit (Nikken Seil Co., Ltd., Shizuoka, Japan) according to the manufacturer’s instructions.

### 4.6. Liver Taurine Content Determination

The liver tissue was homogenized in 100 mM HEPES (pH 7.5), and 4 volumes of 5% sulfosalicylic acid were added to the tissue lysate. After centrifugation, the supernatant was filtered and neutralized with 1 M NaHCO_3_. The samples were subjected to high-performance liquid chromatography (HPLC) to determine the taurine concentration, according to a previously reported method [56]. In brief, the supernatant was derivatized with an OPA reagent (3 mg of o-phthalaldehyde with 50 μL of 95% ethanol, 10 μL of 2-mercaptoethanol in 5 mL of 100 mM borate buffer, pH 10.4) and then subjected to HPLC (D-2000; Hitachi High Technologies, Tokyo, Japan) equipped with a reverse-phase column (Cosmosil 5C18-MS-II, 140 mm; Nacalai Tesque, Kyoto, Japan).

### 4.7. RNA Isolation and Real-Time Polymerase Chain Reaction (PCR)

Total RNA was extracted from liver tissues using Sepasol (Nacalai Tesque) according to the manufacturer’s protocol. cDNA was generated from total RNA by reverse transcription with Rever Tra Ace (Toyobo, Osaka, Japan). Quantitative reverse transcription (RT)-PCR was performed using StepOne (Applied Biosystems, Waltham, MA, USA) with the Thunderbird SYBR qPCR Kit (Toyobo, Osaka, Japan).The primer sequences are listed in Table 2.

### 4.8. Histological and Immunohistochemical Studies

Liver and kidney tissue samples from mice were fixed overnight in 4% paraformaldehyde, followed by dehydration and paraffin infiltration. Embedding and sectioning procedures were then performed to construct 5-μm sections using Leica Microsystems (Wetzlar, Germany). The expression levels of taurine and 8-OHdG were determined in liver and kidneys, and that of 8-OHdG was additionally determined in kidneys by immunohistochemical (IHC) staining. Liver or kidney sections were subjected to dewaxing and rehydration, and antigen retrieval was then performed using a 500-W microwave for 5 min, after which a non-specific binding protein was blocked by incubation in 1% skim milk. After blocking endogenous peroxidase activity by incubation in 1% skim milk for 25 min at room temperature, sections were incubated overnight with a primary antibody mouse monoclonal anti-8-OHdG antibody (5 mg/mL, Japan Institute for the Control of Aging, Shizuoka, Japan) overnight at room temperature in a humid chamber. Following three washes in phosphate-buffered saline (PBS), the sections were subsequently treated with a specific biotinylated secondary antibody and avidin-biotin-peroxidase conjugate (Vector Laboratories, Burlingame, CA, USA). Immunoreaction was visualized by incubation with a DAB peroxidase substrate kit (Nacalai Tesque) and counterstained with hematoxylin in taurine immunostaining for liver. 

The expression levels of insulin and taurine was measured in pancreas tissue samples using double immunofluorescence staining technique. In brief, paraffin-embedded tissues were deparaffinized in xylene and rehydrated in serial alcohol solutions. Antigen retrieval was then performed using a 500-W microwave for 5 min, and non-specific binding protein was blocked by incubation in 1% skim milk. Tissue samples were stained using antibodies against insulin (I2018; SIGMA-ALDRICH, St Louis, MO, USA) and taurine at a 1:400 dilution, overnight. A rabbit polyclonal taurine antibody without a cross-reaction was produced as described previously [57]. After washing with PBS, donkey anti-mouse IgG Alexa Flour 594 secondary antibody (A11011, Invitrogen, Waltham, MA, USA) and donkey anti-rabbit IgG Alexa Flour 488 secondary antibody (A11012; Invitrogen, Waltham, MA, USA) at 1:400 dilution in PBS were incubated for 2 h. Stained tissues were mounted using DAPI-fluoromount-G (Southern Biotech, Birmingham, AL, USA) and observed under a fluorescence microscope (Olympus, Tokyo, Japan). The intensity was evaluated in four different areas of each sample using the ImageJ software program (NIH, Bethesda, MD, USA) for target molecules, and the intensity ratio was calculated in comparison to that of the nuclear staining of DAPI, as a reference for the adjustment of the cell number.

### 4.9. Microarray and Pathway Analyses

Six-week-old male ICR mice were treated with taurine for 8 weeks with 2% (*w/v*) taurine-containing drinking water. RNA from their livers was isolated using Sepazol-RNA Super G (Nacalai Tesque, Kyoto, Japan) and cleaned using an RNeasy Mini Kit (Qiagen GmbH, Hilden, Germany). A microarray analysis was performed on two groups (control and taurine-treated mice, *n* = 3 for each group) using Clariom S arrays (Affymetrix). The array was scanned by using GeneChip^TM^ Scanner 3000 7G (Thermo Fisher Inc., Waltham, MA, USA) and image analysis was performed by using Expression Console^TM^ Software (Thermo Fisher Inc., Waltham, MA, USA). These procedures were performed by Filgen Inc. (Nagoya, Japan). The microarray data are posited in the Gene Expression Omnibus.

### 4.10. Statistics

Results are presented as the means ± standard deviations (S.D.). Comparisons among groups were performed using an analysis of variance and coupled with Dunnett’s tests. All *p*-values less than 0.05 were considered to be significant.

## 5. Conclusions

The present results revealed that taurine supplementation ameliorated hyperglycemia and insulin resistance in STZ-induced type 1 diabetic mice. The hypoglycemic effect of taurine was associated with the improvement of hepatic glucose metabolism, which includes upregulation of the hepatic glucose uptake via GLUT-2 and recovery of the hepatic glycogen content. Suppression of serum ketone body levels by taurine reflects improved hepatic glucose metabolism and energy production. Tissue MDA levels reduced by taurine suggest that the anti-oxidative action of taurine is also involved in the anti-diabetic effect of taurine in STZ-induced diabetic mice.

## Figures and Tables

**Table 1:** Differential expressed genes in the liver of taurine-treated mice identified by microarray analysis.

Gene Symbol	Gene Description	Ratio	*p*-Value
Mt2	metallothionein 2	19.662	0.0077
Aldh1b1	aldehyde dehydrogenase 1 family, member B1	2.893	0.0013
Slc16a7	solute carrier family 16, member 7	2.822	0.0099
Sucnr1	succinate receptor 1	2.767	0.0048
Cyp3a59	cytochrome P450, family 3, subfamily a, polypeptide 59	2.611	0.0097
Ddah1	dimethylarginine dimethylaminohydrolase 1	2.555	0.0055
Hmgn2	high mobility group nucleosomal binding domain 2	2.104	0.0072
Hnmt	histamine N-methyltransferase	2.073	0.0078
Sult1a1	sulfotransferase family 1A, phenol-preferring, member 1	2.025	0.0023
Cyp2j6	cytochrome P450, family 2, subfamily j, polypeptide 6	2.007	0.0015
Siae	sialic acid acetylesterase	1.986	0.0035
Manea	mannosidase, endo-alpha	1.942	0.0090
Papss2	3-phosphoadenosine 5-phosphosulfate synthase 2	1.932	0.0034
Slc6a12	solute carrier family 6	1.926	0.0026
Acss2	acyl-CoA synthetase short-chain family member 2	1.925	0.0004
Slc2a9	solute carrier family 2, member 9	1.816	0.0073
Ech1	enoyl coenzyme A hydratase 1, peroxisomal	0.657	0.0039
Rela	v-rel reticuloendotheliosis viral oncogene homolog A	0.644	0.0089
Ube2m	ubiquitin-conjugating enzyme E2M	0.627	0.0032
H2afz	H2A histone family, member Z	0.623	0.0073
Ldah	lipid droplet associated hydrolase	0.615	0.0055
Mrpl38	mitochondrial ribosomal protein L38	0.579	0.0004
Selk	selenoprotein K	0.578	0.0074
Nfyb	nuclear transcription factor-Y beta	0.576	0.0067
Gucd1	guanylyl cyclase domain containing 1	0.558	0.0066
Mtus1	mitochondrial tumor suppressor 1	0.551	0.0097
Usp6nl	USP6 N-terminal like	0.544	0.0002
Cd9	CD9 antigen	0.543	0.0028
Lurap1l	leucine rich adaptor protein 1-like	0.535	0.0008
Gpr182	G protein-coupled receptor 182	0.533	0.0078
Paqr9	progestin and adipoQ receptor family member IX	0.530	0.0023
Arpc1b	actin related protein 2/3 complex, subunit 1B	0.520	0.0001
Inhbc	inhibin beta-C	0.508	0.0087
Crem	cAMP responsive element modulator	0.482	0.0019
Txndc5	thioredoxin domain containing 5	0.453	0.0054
Gm10181	predicted gene 10181	0.442	0.0074
Myc	myelocytomatosis oncogene	0.247	0.0016
Csad	cysteine sulfinic acid decarboxylase	0.041	0.0048

**Table 2:** Primer sequences uded in the experiment.

Gene	Forward Primer	Reverse Primer
GLUT-2	TTCATGTCGGTGGGACTTGTG	TGGCAGTCATGCTCACGTAACT
G-6-P	AACGTCTGTCTGTCCCGGATCTAC	TTCCGGAGGCTGGCATTGTA
GK	GTACGACCGGATGGTGGATG	TCTACCAGCTTGAGCAGCAC
PEPCK	CGAATGTGTGGGCGATGAC	ACTGAGGTGCCAGGAGCAACT
SREBP1c	AATGACAAGATTGTGGAGCTCAAAG	ACACCAGGTCCTTCAGTGATT

## Data Availability

The data presented in this study are available in the article.

## Abbreviations

GK	Glucokinase
GLUT-2	Glucose transporter-2
G6Pase	Glucose-6-phosphatase
MDA	Malondialdehyde
8-OHdG	8-Hydroxy-2′-deoxyguanosine
PEPCK	Phosphoenolpyruvate carboxykinase
ROS	Reactive oxygen species
SREBP-1c	Sterol regulatory element-binding protein 1c
STZ	Streptozotocin
